# External control of fluoridation in the public water supplies of Brazilian cities as a strategy against caries: a systematic review and meta-analysis

**DOI:** 10.1186/s12903-021-01754-2

**Published:** 2021-08-19

**Authors:** Bárbara Souza Martins Rosário, Henrique Damian Rosário, Walbert de Andrade Vieira, Graziela Oro Cericato, Diego Figueiredo Nóbrega, Cauane Blumenberg, Fernando Neves Hugo, Márcio Magno Costa, Luiz Renato Paranhos

**Affiliations:** 1 Pharmacology School, Universidade do Sul de Santa Cantarina (UNISUL), Tubarão, SC Brazil; 2 Department of Dentistry, Dental School, Universidade do Sul de Santa Cantarina (UNISUL), Tubarão, SC Brazil; 3 Endodontics Division, Department of Restorative Dentistry, School of Dentistry of Piracicaba, Universidade Estadual de Campinas (UNICAMP), Piracicaba, SP Brazil; 4 Dental School, Faculdade Meridional (IMED), Passo Fundo, RS Brazil; 5 Professional Masters Research in Health, Centro Universitário CESMAC, Maceió, AL Brazil; 6 Department of Social Medicine, Universidade Federal de Pelotas (UFPel), Pelotas, RS Brazil; 7 Department of Preventive and Social Dentistry, Universidade Federal do Rio Grande do Sul (UFRGS), Porto Alegre, RS Brazil; 8 Division of Department of Removable Prosthesis and Dental Materials, School of Dentistry, Universidade Federal de Uberlândia (UFU), Uberlândia, MG Brazil; 9 Division of Preventive and Community Dentistry, School of Dentistry, Universidade Federal de Uberlândia (UFU), Campus Umuarama, Av. Pará, 1720, Bloco 2G, sala 1, Uberlândia, MG 38405-320 Brazil

**Keywords:** Fluoridation, External control, Water treatment

## Abstract

**Background:**

Among the methods currently available to provide fluoride in population levels, fluoridated water is the most successful for presenting high efficacy, safety and good cost–benefit. However, recent studies on external control have shown great variability of fluoride concentrations in the water from treatment stations in Brazilian cities, which must present concentration between 0.6 and 0.8 mg/L to be considered acceptable in most cities. Thus, this study aimed to perform a systematic review of the literature to assess the adequacy of fluoride concentration in the water in Brazilian cities using external control.

**Methods:**

The protocol was registered in PROSPERO. Six databases were used as primary search sources and three databases were used to partially capture the "gray literature". Only observational studies that assessed the fluoride concentration of artificially fluoridated water from the public supply network were included. The JBI Critical Appraisal Tools for Systematic Reviews was used to assess the risk of bias of the studies. A proportion meta-analyses using random-effect models were performed. The heterogeneity between studies was determined by I^2^ statistic. Meta-regressions were conducted to identify relevant moderators to be used in stratified meta-analyses. Publication bias was investigated by Egger’s tests.

**Results:**

The search provided 2038 results, from which 14 met the eligibility criteria and were included in the data extraction of the review. Overall, the water samples were collected from 449 different sources in three Brazilian regions. Thirteen studies presented a low risk of bias. The mean concentration of fluoride ranged from 0.17 to 0.89 ppmF. The meta-analyis demonstrated that more than half of the water samples analyzed had fluoride concentration levels outside the acceptable range (56.6%; 95% CI 45.5; 67.3), with high heterogeneity.

**Conclusion:**

More than half of the public water supply analyzed in the studies selected had fluoride concentration levels outside the acceptable range, which may affect the risk of developing oral diseases in the Brazilian population, having an important impact on public health.

**Supplementary Information:**

The online version contains supplementary material available at 10.1186/s12903-021-01754-2.

## Background

In 1942, it was found that natural fluoride in public water supplies could reduce the prevalence of dental caries in different populations [1]. Thereafter, the scientific evidence constructed over the following decades recognized fluoride as the most successful agent used for caries prevention and control in Dentistry [2–5]. Several systematic reviews show clear evidence that the presence and addition of fluoride in the water [2], dentifrices [3], gels [5], or varnishes [4] can reduce the prevalence of dental caries.

Among the methods currently available to provide fluoride in population levels, fluoridated water is the most successful for presenting high efficacy, safety [2] and good cost–benefit [6]. Moreover, the addition of fluoride in the water supply is considered a socially equitable measure because it benefits the entire population with access to piped water [7].

The addition of fluoride in the public water supply was initially proposed in 1945 by the United States and since then it has been recommended by the World Health Organization as a key strategy to prevent dental caries [8]. Brazil has the second largest fluoridation system for public water supplies in the world, only after the United States [9]. However, different from the United States, Brazil does not have a national surveillance system and although adding fluoride in the public water supply is mandatory since 1974 [10], it is estimated that only 50% of the Brazilian population has access to fluoridated water [11].

Furthermore, fluoride can occur naturally in water. The percentage of the Brazilian population supplied with naturally fluoridated water is unknown. However, the report of moderate and severe fluorosis (associated with the consumption of water containing natural fluoride in high concentrations) is rare and generally associated to the consumption of groundwater [12]. In regions where fluoride occurs naturally, the Brazilian Ministry of Health defines the value of 1.5 ppm F as the maximum allowed limit [13].

The benefit of fluoridation in public water supplies depends on the continuity of such measure over time and on the regular maintenance of "optimal" fluoride concentrations in the water [14]. Optimal concentration is one that can produce the maximum benefit in caries prevention and control combined with a minimum risk of developing dental fluorosis [15]. The optimal content of fluoride may vary according to temperature, location, and volume of water ingestion, but most Brazilian cities use values between 0.6 and 0.8 ppm [16]. Therefore, it is imperative that besides the operational control performed by the sanitation company in charge there is an external control of the quality of water provided to consumers, performed by an independent institution, not involved in the fluoridation process [14]. Recent results of individual studies on external control have shown great variability of fluoride concentrations in the water from treatment stations in Brazilian cities [17–20], which may compromise the benefit of this major public health measure on caries control or even cause dental fluorosis, when fluoride concentration is below or above the optimum, respectively.

Thus, this study aimed to perform a systematic review of the literature to assess the adequacy of fluoride concentration in the artificially fluoridate water in Brazilian cities using external control.

## Methods

### Protocol and registration

The protocol was registered at the International Prospective Register of Systematic Reviews (PROSPERO) database, under number CRD42019120870 (http://www.crd.york.ac.uk/PROSPERO). This systematic review was reported according to the Preferred Reporting Items for Systematic Reviews and Meta-Analyses (PRISMA) [21] and conducted according to the Joanna Briggs Institute (JBI) Manual [22].

### Study design and eligibility criteria

This study is based on the following research question: “Does the public water supply systems from Brazilian cities provide water with ideal fluoride concentrations?”.

This systematic review included cross sectional studies reporting on the external control of artificial fluoridation of the public water supply in Brazilian cities and the frequency of water samples with fluoride levels within or outside the acceptable range, without restrictions of year or language. The inclusion criteria included studies that analyzed at least one water sample per month for 12 months [14]. Moreover, only studies using the electrometric method to measure fluoride concentration were selected. This method uses a specific electrode for fluoride ion coupled to a potentiometer. The electrode is calibrated with standard fluoride solutions to allow measuring and comparing the concentration of fluoride in water samples in a proper standard curve [23]. The ion-specific electrode presents accurate and fast results, and it is considered the "gold standard" for this type of analysis [24].

The exclusion criteria were: (1) studies outside the objective; (2) literature reviews, case reports, letters to the editor, editorials, indexes, abstracts, and reports; (3) studies using secondary data sources; (4) studies that did not use water from a public supply; (5) studies that used water from naturally fluoridated sources.

### Sources of information and search

The Embase, Latin-American and Caribbean Health Sciences Literature (LILACS), MedLine (via PubMed), SciELO, Scopus, and Web of Science databases were used as primary study sources. OpenThesis, OpenGrey, and OATD were used to partially capture the "gray literature". A manual search was also performed through a systematized analysis of the references of the eligible articles. All steps were performed to minimize selection and publication biases.

The following MeSH descriptors were used: "Drinking Water", "Water Supply", "Quality Control", “Brazil”. In addition, the following synonyms and free terms were used to enhance the search: “Potable Water”, “public water supply”, “Water fluoridation”, “monitoring”, "External Control", "Fluoride concentration", “Operational control”, “Brazilian”. The Boolean operators "AND" and "OR" were used to enhance the search strategy through several combinations (Table 1). The search strategies were adapted for each database respecting their rules of syntax. The bibliographic search was conducted until August 10, 2020.

**Table 1:** Strategies for database search

Database	Search Strategy (August, 2020)
*Main database*	
**MedLine (via PubMed)** http://www.ncbi.nlm.nih.gov/pubmed	(“Drinking Water" OR "Drinking Water" OR "Potable Water" OR “public water supply” OR “Water fluoridation” OR “Water supply”) AND (“Quality Control” OR “monitoring” OR “External Control” OR “Fluoride concentration” OR “Operational control”) AND (“Brazil” OR “Brazilian”)
**Scopus** http://www.scopus.com/	( ( "Drinking Water" OR "Potable Water" OR "public water supply" OR "Water fluoridation" OR "Water supply") AND ( "Quality Control" OR "monitoring" OR "External Control" OR "Fluoride concentration") AND ( "Brazilian" OR "Brazil"))
**LILACS** http://lilacs.bvsalud.org/	tw:( "Drinking Water" AND “External Control”) AND (instance:"regional") AND (db:("LILACS"))
	tw:( “public water supply” AND “External Control”) AND (instance:"regional") AND (db:("LILACS"))
	tw:(“Water fluoridation” AND “External Control”) AND (instance:"regional") AND (db:("LILACS"))
	tw:(“Fluoretação da água” AND “Heterocontrole”) AND (instance:"regional") AND (db:("LILACS"))
	tw:(“Fluoreto” AND “Água de abastecimento”) AND (instance:"regional") AND (db:("LILACS"))
	tw:(“public water supply” AND “Fluoride content monitoring”) AND (instance:"regional") AND (db:("LILACS"))
	tw:(“water for human consumption” AND “Fluoride”) AND (instance:"regional") AND (db:("LILACS"))
**SciELO** http://www.scielo.org/	Drinking Water AND External Control
	Public water supply AND External Control
	Water fluoridation AND External Control
	Fluoretação da água AND Heterocontrole
	Fluoreto AND Água de abastecimento
	Public water supply AND Fluoride content monitoring
	Water for human consumption AND Fluoride
**Embase** http://www.embase.com	('drinking water'/exp OR 'drinking water' OR 'potable water'/exp OR 'potable water' OR 'public water supply' OR 'water fluoridation'/exp OR 'water fluoridation' OR 'water supply'/exp OR 'water supply') AND ('quality control'/exp OR 'quality control' OR 'monitoring'/exp OR 'monitoring' OR 'external control' OR 'fluoride concentration') AND ('Brazilian'/exp OR 'Brazilian' OR 'brazil'/exp OR 'brazil')
**Web Of Science** http://apps.webofknowledge.com/	((“Drinking Water" OR "Drinking Water" OR "Potable Water" OR “public water supply” OR “Water fluoridation” OR “Water supply”) AND (“Quality Control” OR “monitoring” OR “External Control” OR “Fluoride concentration” OR “Operational control”) AND (“Brazil” OR “Brazilian”))
*Grey literature*	
**OpenGrey** http://www.opengrey.eu/	("Water fluoridation” OR “public water supply”) AND (“External Control” OR “Quality Control”)
	("Fluoridation" OR "Fluorides") AND ("Water Quality Control" OR "Water monitoring")
**OpenThesis** http://www.openthesis.org/	("Fluoridation" OR "Fluorides") AND ("Water Quality Control" OR "Water monitoring")
	("Water fluoridation” OR “public water supply”) AND (“External Control” OR “Quality Control”)
**Open Access** **Theses and Dissertations (OATD)** https://oatd.org/	("Fluoridation" OR "Fluorides") AND ("Water Quality Control" OR "Water monitoring")
	("Water fluoridation” OR “public water supply”) AND (“External Control” OR “Quality Control”)

The results obtained were exported to the EndNote Web™ software (Thomson Reuters, Toronto, Canada), in which duplicates were removed automatically. The remaining results were exported to Microsoft Word™ 2010 (Microsoft™ Ltd, Washington, USA), in which the remaining duplicates were removed manually.

### Study selection

A calibration exercise was performed before the selection phases, in which the reviewers discussed the eligibility criteria and applied them to a sample of 20% of the studies retrieved to determine inter-examiner agreement (Kappa ≥ 0.81). At all phases, two eligibility reviewers (HDR and WAV) performed the readings independently, and any disagreements between inter-examiners were discussed with a third reviewer (LRP) to reach a consensus. The first phase consisted of a methodical analysis of the titles of the studies. In the second phase, the abstracts were read for the initial application of the exclusion criteria. The titles that met the objectives of the study but did not have abstracts available were fully analyzed in the third phase, in which preliminary eligible studies had their full texts obtained and evaluated to verify whether they fulfilled the eligibility criteria. The excluded studies were registered separately, explaining the reasons for exclusion (Additional file 1: Table S1).

### Process of data collection and extraction

The studies were analyzed and the following data were extracted: study identification (author, year, city, and mean annual temperature of the city assessed), characteristics of the collected water samples (collection sites, approximate volume collected, time of collection, frequency of collection, number of samples, and value of fluoride used as parameter), and specific results (mean fluoride concentration, standard deviation of fluoride concentration, and samples within and outside the standard used). In cases of studies with incomplete data, an e-mail was sent to the corresponding author to gather the information. The frequency of samples within or outside the acceptable range was calculated when not presented in the original article.

To ensure the consistency among reviewers, a calibration exercise was performed with both reviewers (HDR and WAV), in which information was extracted jointly from an eligible study. Any disagreement between the reviewers was solved through discussions and when both reviewers could not agree, a third one (LRP) was consulted to make a final decision.

### Risk of bias of studies

The "JBI Critical Appraisal Tools for use in JBI Systematic Reviews" [25] assessed the risk of bias of the studies. Two calibrated authors (WAV and DFN) assessed independently each domain regarding their potential risk of bias; in case of disagreement, a third reviewer (LRP) was consulted to make a final decision. Each study was categorized according to the rate of positive answers.

This tool is composed of nine questions, as follows: (1) Was the sample frame appropriate to address the target population? (2) Were study participants sampled in an appropriate way? (3) Was the sample size adequate? (4) Were the study subjects and the setting described in detail? (5) Was the data analysis conducted with sufficient coverage of the identified sample? (6) Were valid methods used for the identification of the condition? (7) Was the condition measured in a standard, reliable way for all participants? (8) Was there appropriate statistical analysis? (9) Was the response rate adequate, and if not, was the low response rate managed appropriately?

Each item could be answered as: “yes”—if the study did not present bias regarding the domain evaluated by the question; or “no”—if the study presented bias regarding the domain evaluated by the question; or “unclear”—if the study did not provide sufficient information to evaluate the bias in the question; or (4) “Not Applicable”—if the question was not suitable for the study.

Risk of bias was considered *High* when the study obtained 49% of "yes" answers, *Moderate* when the study obtained 50–69% of "yes" answers, and *Low* when the study reached more than 70% of "yes" answers [26].

### Statistical analyses

The main outcome analyzed was the frequency of water samples with fluoride levels outside the acceptable range. According to the Ministry of Health of Brazil, the ideal levels of fluoride concentration in the water supply will depend on the annual average temperature of the fluoridation site. As Brazil is a country of continental dimensions, the climate is not homogeneous throughout the country; consequently, optimal fluoride levels also vary from region to region. For this reason, each eligible study adopted different acceptability ranges.

The overall prevalence was estimated for each study by adding the number of samples below and above the boundaries of the acceptable fluoride range adopted in each eligible study and dividing it by the total number of water samples collected. We also calculated the frequency of water samples with fluoride levels exclusively below and above the acceptable range adopted in each eligible study.

Proportion meta-analyses using random-effect models were conducted to estimate the combined prevalence of water samples with fluoride levels outside the acceptable range adopted in each eligible study. Similar meta-analytical models were fitted to evaluate the prevalence of water samples exclusively below and above the acceptable range. The Freeman-Tukey double arsine transformation was applied to stabilize variances and to include zero and 100% prevalence in the estimation of the pooled effects [27]. The heterogeneity between studies was assessed with I^2^ statistic. Publication bias was investigated with Egger’s test for overall and stratified meta-analysis.

The following moderators were individually meta-regressed on the outcome to assess their influence on the between-study heterogeneity: acceptable range adopted by the study (0.6–0.8 ppmF, other), Brazilian region where the samples were collected, type of water fluoridation, mean annual temperature, average number of samples collected per month, total number of samples collected, total number of sample collection sites, and volume of water collected in each sample. A 20% significance (*p* < 0.2) level was adopted to consider a moderator as being relevant. In this case, a stratified meta-analysis according to the levels of the moderator was conducted.

After meta-regressing the statistically relevant moderators on the pooled prevalence, the diagonal values of the hat matrix were calculated and plotted against the prevalence of each study in order to identify outliers or leverage points. As a sensitivity analysis, we removed outliers or leverage studies to evaluate how this could influence the estimates. All statistical analyses were performed using the Stata 16 software (StataCorp, College Station, TX, USA).

## Results

### Study selection

**Fig. 1:**
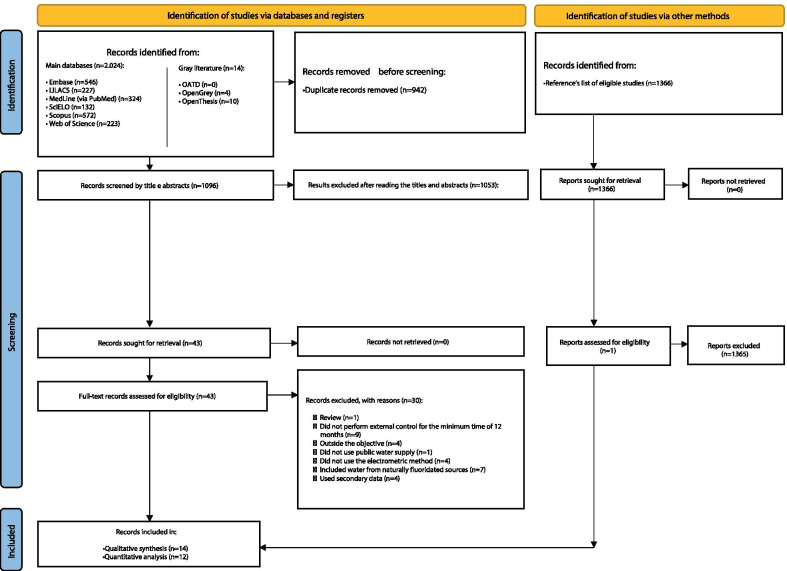
Flowchart of the process of literature search and selection, adapted from the PRISMA statement

During the first phase of study selection, 2038 results were found, including the "gray literature". After analyzing the titles and abstracts, 43 articles were eligible for the full-text analysis. The manual searching through the references of the eligible articles found one article. Thus, from the 44 studies selected in this phase, only 14 studies [17, 28–40] continued for the qualitative analysis of results (Fig. 1). Two of these studies [28, 36] were not considered in the quantitative analyses because the results were given by the number of collection sites and not by the number of samples.

### Characteristics of the studies

Overall, the external control was conducted in 42 cities from three Brazilian regions: four of them in the northeast region [28–30], thirty-two in the southeast region [30–34], and six in the south region [17, 36–40] (Table 2). São Paulo and Rio Grande do Sul were the most studied Brazilian federations units, with 3 studies each [32–34, 36, 37, 39]. The average annual temperature of the cities varied between 17.9 and 28.4 °C.

**Table 2:** Summary of the main characteristics of the eligible studies

Author, year [ref]	City of collection	Mean annual temperature °C (Variation)	Collection sites	Number of collection sites	Approximate volume collected (by sample)	Time of collection	Number of samples collected per month
Maia et al. [31]	Niterói/RJ	28.4 °C	Directly at the treatment station	1	+	January 2000–December 2000	2
Lima et al. [36]	Pelotas/RS	+	Public locations	16	5 mL	November 1999–October 2001	1
Moura et al. [26]	Teresina/PI	27 °C	Public locations	5	5 mL	June 2000–May 2001	1
Piva et al. [37]	Cachoreira do Sul/RS	+	Basic Health Units or locations closer to the treatment unit	8	80 mL	April 2005–March 2006	1
Amaral et al. [32]	Piracicaba/SP	(26.3–32.5 °C)	Daycares in all the regions of the city	35	5 mL	April 2004–February 2006	1
Silva et al. [30]	Teresina, Floriano, and Parnaíba/PI	28 °C	Basic Health Units and water treatment station	18 (six per city)	2 mL	2004–2005	1
Panizzi et al. [38]	Chapecó/SC	+	Basic Health Units	10	50 mL	November 1995–November 2005	1
Saliba et al. [33]	8 cities northeast the state of São Paulo^⁋^	(26.3–32.5 °C)	Public locations	24 (three per city)	+	November 2004–October 2007	1
Peixoto et al. [29]	Jaguaribara/CE	(26.3–32.5 °C)	Two public and one particular institution	3	15 mL	August 2010–July 2011	2
Moimaz et al. [34]	29 cities in São Paulo^⁋⁋^	(26.3–32.5 °C)	Public locations	193	+	November 2004–October 2008	1
Assaf et al. [35]	Nova Friburgo/RJ	27.9 °C	Public institutions	60	50 mL	March 2004–February 2011	1
Bergamo et al. [17]	Maringá/PR	17.9 °C	Site close to the treatment station	26	100 mL	December 2010–November 2011	1
Brito et al. [39]	Passo Fundo/RS	27.99 °C	Basic Health Units	28	5 mL	July 2009–July 2010	1
Kuhnen et al. [40]	Lages/SC	18.1 °C	*	22	+	January 2013–December 2013	1

+ Not mentioned by the author; ^⁋^Alto Alegre, Bento de Abreu, Coroados, Gabriel Monteiro, Lourdes, Piacatu, Sud Mennucci, and Turiúba; ^⁋⁋^Alto Alegre, Araçatuba, Auriflama, Bento de Abreu, Birigui, Brejo Alegre Castilho, Coroados, Gabriel Monteiro, Guaraçai, Guararapes, Guzolândia, Ilha Solteira, Itapura, Lavínia, Lourdes, Mirandópolis, Murutinga do Sul, Nova Castilho, Nova Independência, Nova Luzitânia, Penápolis, Piacatú, Rubiácea, Santópolis do Aguapeí, Sud Mennucci, Suzanápolis, Turiúba,Valparaíso

Water samples were collected between 1995 and 2013 and the length of external control varied between 1 and 10 years. All studies [17, 28–40] collected at least one water samples every month. A total of 449 sites were used to collect water samples, including schools, clinics and hospitals, public institutions, or directly from the water treatment station.

### Risk of bias of studies

Thirteen studies [17, 29–40] presented a low risk of bias, and one study [28] presented a moderate risk of bias (Table 3). Item 9 of the assessment instrument was considered 'not applicable' for not presenting the possibility of dropouts throughout the studies.

**Table 3:** Risk of bias assessed by the “The Joanna Briggs Institute Critical Appraisal tools for use in JBI Systematic Reviews”

Study [ref]	Q.1	Q.2	Q.3	Q.4	Q.5	Q.6	Q.7	Q.8	Q.9	% yes/risk
Maia et al. [31]	√	√	√	√	√	√	√	√	N/A	100%/low
Lima et al. [36]	√	U	√	--	√	√	√	√	N/A	75%/low
Moura et al. [26]	--	U	--	√	√	√	√	√	N/A	62.5%/moderate
Piva et al. [37]	√	√	√	√	√	√	√	√	N/A	87.5%/low
Amaral et al. [32]	√	--	√	√	√	√	√	√	N/A	75%/low
Silva et al. [30]	√	√	--	√	√	√	√	√	N/A	100%/low
Panizzi et al. [38]	√	U	√	√	--	√	√	√	N/A	75%/low
Saliba et al. [33]	√	√	--	√	√	√	√	√	N/A	75%/low
Peixoto et al. [29]	--	--	√	√	√	√	√	√	N/A	75%/low
Moimaz et al. [34]	--	√	--	√	√	√	√	√	N/A	75%/low
Assaf et al. [35]	√	--	√	√	√	√	√	√	NA	87.5%/low
Bergamo et al. [17]	√	--	√	√	√	√	√	√	N/A	87.5%/low
Brito et al. [39]	√	U	--	--	√	√	√	√	N/A	75%/low
Kuhnen et al. [40]	√	U	√	√	√	√	√	√	N/A	87.5%/low

Q.1—Was the sample frame appropriate to address the target population? Q.2—Were study participants recruited in an appropriate way? Q.3—Was the sample size adequate? Q.4—Were the study subjects and setting described in detail? Q.5—Was data analysis conducted with sufficient coverage of the sample identified? Q.6—Were valid methods used to identify the condition? Q.7—Was the condition measured in a standard and reliable way for all participants? Q.8—Was there appropriate statistical analysis? Q.9—Was the response rate adequate, and if not, was the low response rate managed appropriately? √—Yes; --—no; U—unclear; NA—not applicable

### Individual results of eligible studies

Most studies considered 0.6–0.8 ppmF as an acceptable range of fluoride levels [28–34]. The mean fluoride concentration varied from 0.17 to 0.89 ppmF. Nine studies [17, 28, 29, 32–34, 36, 37, 40] found that most of the sample were within the acceptable range and five found that most of the samples were outside the acceptable range [30, 31, 35, 38, 39]. The percentage of the samples within, below, and above the acceptable range varied from 4 to 86%, 2.86% to 95.7%, and 0% to 45%, respectively (Table 4).

**Table 4:** Summary of the main individual results of the eligible studies included in the qualitative analysis that used only artificial fluoridated water

Study [ref]	Total samples (n)	Value of F used as parameter (in ppmF)	Mean fluoride concentration in the period (in ppmF)	Standard deviation of fluoride concentration (in ppmF)	Samples within the standard used (n/%)	Samples outside the standard used (n/%)
Maia et al. [31]	48	0.6–0.8	0.45	+	2 (4%)	< 0.6 ppm = 30 (62.5%) > 0.8 ppm = 16 (33.5%)
Lima et al. [36]	764	0.6–0.9	0.68	+	8 (50%)**	8 (50%)^#^
Moura et al. [26]	180	0.60–0.80	0.623	0.168	32 (53.3%)**	< 0.6 ppm = 22 (36.7%)^#^ > 0.8 ppm = 6 (10%)^#^
Piva et al. [37]	104	0.6–0.9	0.66	+	66 (61.1%)	38 (38.9%)^##^
Amaral et al. [32]	630	0.6–0.8	0.7	+	535 (84.92%)	< 0.6 ppm = 18 2.86%) > 0.8 ppm = 77 (12.2%)
Silva et al. [30]	576	0.6–0.8	0.24 (Teresina) 0.27 (Floriano) 0.17 (Parnaíba)	0.07 (Teresina) 0.06 (Floriano) 0.03 (Parnaíba)	25 (4.3%)	< 0.6 ppm = 551 (95.7%) > 0.8 ppm = 0
Panizzi et al. [38]	989	0.7–1.0 (I) 0.7–0.9 (II) 0.65–0.94 (III)	0.89	+	455 (46%) 316 (32%) 425 (43%)	534 (54%)^##^ 673 (68%)^##^ 564 (57%)^##^
Saliba et al. [33]	864	0.6–0.8	+	+	669 (77.4%)	< 0.6 ppm = 171 (13.5%) > 0.8 ppm = 24 (2.7%)
Peixoto et al. [29]	72	0.6–0.8 (I) 0.55–0.84 (II)	0.55	0.19	I—34 (47.2%) II—46 (63.9%)	I—< 0.6 ppm = 32 (44.4%) > 0.8 ppm = 6 (8.3%) II—< 0.55 ppm = 26 (36.1%) > 0.84 ppm = 0
Moimaz et al. [34]	6862	0.6–0.8	0.64	0.28	3671 (53.5%)	< 0.6 ppm = 2084 (30.4%) > 0.8 ppm = 1107 (16.1%)
Assaf et al. [35]	179^⁋^	0.65–0.94	+	+	87 (48.6%)	< 0.65 ppm = 92 (51.4%) > 0.94 ppm = 0
Bergamo et al. [17]	252^⁋^	0.55–0.84	0.77	+	217 (86%)	35 (14%)^##^
Brito et al. [39]	121	0.6 a 0.9 (I) 0.65 a 0.94 (II)	0.57	0.12	I—48 (39.7%) II—26 (21.4%)	I—< 0.6 ppm = 73 (60.3%) > 0.9 ppm = 0 (0%) II—< 0.65 ppm = 95 (78.6%) > 0.94 = 0 (0%)
Kuhnen et al. [40]	737	0.7 a 1.0 (I) 0.65 a 0.94 (II)	+	+	I—432 (58.6%) II—377 (51.2%)	I—< 0.7 ppm = 49 (6.7%) > 1.0 ppm = 256 (34.7%) II—< 0.65 ppm = 29 (3.8%) > 0.94 = 331 (45%)

+ Not mentioned by the author

^#^The result was given by the number of collection sites and not by the number of samples

^##^Did not separate between < 0.6 or > 0.9

^⁋^The data extracted referred only to the water artificially supplemented with fluoride

### Meta-regression and analyses

**Fig. 2:**
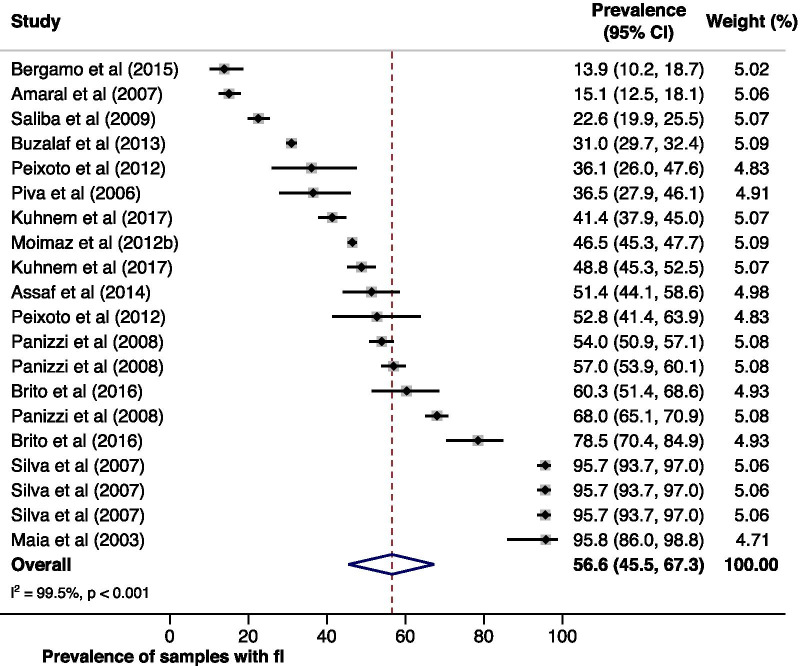
Overall prevalence of samples with fluoride concentrations outside the acceptable range and according to each study

The frequency of water samples with fluoride concentration outside the acceptable range varied between 13.9% and 95.8% (Fig. 2). Grouping all estimates, the pooled prevalence was 56.6% (95% CI 45.5; 67.3). However, there was high heterogeneity between the studies (I^2^ = 99.5%). After meta-regressing all moderators on the pooled prevalence, the only relevant moderator at 20% significance level was the Brazilian region where the study was conducted. Thus, a stratified meta-analysis was conducted according to the levels of this moderator (Additional file 2: Table S2).

**Fig. 3:**
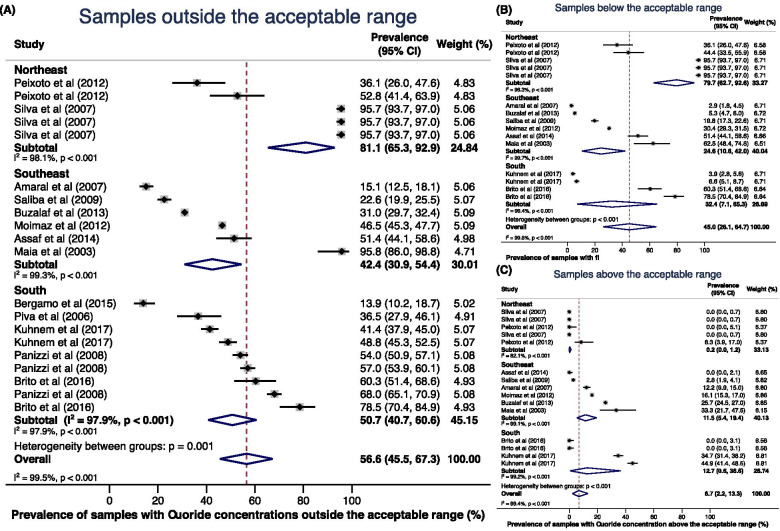
Prevalence of samples with fluoride concentrations **A** outside the acceptable range, **B** below the acceptable range, and **C** above the acceptable range, according to the Brazilian region

The Northeast was the Brazilian region with the highest prevalence of water samples with fluoride concentration outside the acceptable range, with 81.1% (95% CI 65.3; 92.9) (Fig. 3A). The combined estimate revealed that, in the Northeast Brazilian region, 79.7% of the samples were below the acceptable range (Fig. 3B) and only 0.2% were above the acceptable range (Fig. 3C). The Southeast region had the lowest prevalence (42.4%; 95% CI 30.9; 54.4), with 24.6% of the sample below the acceptable range and 11.5% above it.

We estimated the diagonal values of the hat matrix to identify studies that might be outliers or influential for the pooled estimates (Additional file 3: Fig. S1). The study by Silva et al. [30] presented the highest leverage values, thus it was removed from the meta-analytical model as a sensitivity analysis. By doing so, the pooled prevalence was 9.5 percentage points lower compared to the analysis considering all studies, but without statistical significance (Pooled prevalence = 47.1%; 95% CI 39.6; 54.7). In turn, the between-study heterogeneity reduced to 98.9%

There was no evidence of publication bias for the overall effects of the meta-analyses with samples outside, below or above the acceptable range. The only evidence of bias for the models analyzing samples outside the acceptable range was for the Northeast region, while for samples below the acceptable range there was evidence of bias for the Northeast and South regions (Additional file 2: Table S2).

## Discussion

Although water fluoridation in Brazil has been mandatory since the 70s [9], in practice, millions of Brazilians do not have access to this important preventive measure. The epidemiological consequences of these acts were highlighted in last oral health survey carried out in Brazil [41], which showed that 54% of the 5-year-old children had experienced caries. The caries experience increases with age, reaching impressive 27.5 decayed, missing or filled teeth (DMF-T) among the elderly. In this context, water fluoridation remains an effective measure for caries prevention, considering that in regions highly covered by water fluoridation, like South and Southeast, the mean DMF-T (2.06 and 1.72, respectively) was considerably lower than that found in regions with low coverage of fluoridated water, such as North and Northeast (mean DMF-T = 3.16 and 2.63, respectively).

The preventive effects of fluoride are more efficient when associated with water consumption because it is a vehicle of high ingestion and frequency, collective coverage, and low cost [6]. Several countries that perform water fluoridation observed a drop in caries indexes after implementing such measure [42, 43]. Every time fluoridated water or food cooked with it is ingested, a transient increase of fluoride concentrations in saliva and dental biofilm is observed (lasts about 1 to 2 h). Subsequently, fluoride is absorbed and distributed to the body. Part of the absorbed fluoride returns to the oral cavity through saliva. Thus, the frequent and continuous intake of fluoridated water maintains high fluoride levels in the oral cavity, to interfere with the processes of de- and remineralization [44].

Our results showed that less than half of the total water samples were within the standards established by law [16]. Although guaranteed by law, the surveillance of fluoridation in Brazilian public water supplies is deficient and may be attributed to factors such as local temperature, instability of the fluoride salt, or even to the difficulty regarding the functioning of the supply system [11].

Another result observed in the present meta-analysis was the high number of samples below the minimum concentration required to guarantee the benefits of fluorided water in caries control. The clinical effect of this variation will depend on the exposure duration. When fluoride is used in a community-based approach like fluoridated water, the maintenance of the optimal fluoride concentration is essential to guarantee its effect on caries control. If fluoridated water is constantly ingested in doses below the “optimal”, the retention of fluoride in oral fluids (saliva and biofilm fluid) will be low, compromising the preventive and therapeutic effect of this measure on caries development. The cities with the best indexes of fluoridation are in the southeast region and the worst indexes are in the northeast region. Such finding may reflect the different levels of social development of these regions, considering that a great portion of the states in the northeast region present the lowest human development index (HDI) of the country [45]. This result is supported by Daré et al. [46], in which the regions with the lowest HDI presented the worst results in the fluoridation of the water supply. Thus, the awareness for better training and control of the technical fluoridation team should be performed by government agencies to provide the whole population with ideal fluoridation levels in public water supplies.

The systemic chronic ingestion of fluoride is related to dental fluorosis [47]. Fluorosis is described as a symmetrical hypomineralization that affects tooth enamel. In the present study, 6.7% of the samples presented fluoride concentrations above the recommendation. Considering that the included studies assessed the fluoride concentration in water for at least 12 months, and in some cases the fluoride concentration was maintained elevated for consecutive months, the daily consumption of this water or food prepared with it may lead to the occurrence of dental fluorosis. However, the clinical effect of this fluorosis does not appear to be a concern, since the last Brazilian dental survey found a 16% prevalence of dental fluorosis in 12 year-old children, which was restricted to mild and very mild severity forms [48].

Besides drinking fluoridated water, these children also used fluoridated toothpaste daily. In fact, studies conducted in countries where the population is exposed to these two sources of fluoride, such as United States and Australia, show that dental caries affects a person’s quality of life more than fluorosis [49, 50]. This can be explained by the fact that the most common levels of fluorosis registered in these countries are mild and very mild, similarly to what is observed in Brazil.

Furthermore, early access to fluoride products, including the use of fluoridated toothpaste, use of mouthwash solutions and professional application before the age of three are also pointed out as a risk factors for dental fluorosis. In addition, other additional sources of fluoride such as mineral water, fluoridated salt, teas and children's drinks can increase the risk of dental fluorosis in children. In the Brazilian context, however, fluoridated salt is not available [51]. Thus, considering that fluoridated water and the use of fluoridated toothpaste are the most efficient and cost-effective strategies to prevent caries, other forms of delivery should be indicated only for people at high risk for caries or disease activity. A recent article showed that dental caries experience in children was related with the effectiveness and frequency of oral hygiene and diet [52]. Caries prevention programs must be adjusted to individual characteristics of each child, taking into consideration oral hygiene practices, diet and total fluoride intake [52].

It is worth noting that external control is only a mechanism to detect the problem of water fluoridation and not the solution. Public policies to guarantee ideal levels of fluoridation need to be implemented, with stricter enforcement of the law. Moreover, to ensure the benefits of fluoride and minimize the risk of dental fluorosis, it is recommended that caries community prevention programs take into consideration the estimate of total fluoride exposure from water or food prepared with it, dentifrices and mouthrinses, as well as the oral hygiene and dietary habits of the studied population [52].

Many countries have policies to maximize the benefits of fluoride, but many have yet to do so. Policies were introduced to reduce excessive fluoride exposure during the period of tooth development, and these were successful in reducing dental fluorosis without compromising caries prevention [53]. In Brazil, water fluoridation has been provided by law since 1975, with varying degrees of implementation throughout the country. Historically, richer, more developed regions benefited from water fluoridation earlier, however the Brazilian Oral Health Policy of 2004 promoted fluoridation of more deprived areas, resulting in the reduction of inequalities in access to fluorides. Thus, even considering that the topical application of fluoride, especially brushing with fluoride toothpaste, is the most important preventive procedure that maintains the cariostatic concentration of fluor in the oral environment, the fluoridation of water at ideal levels of fluoride concentration represents an important public health action, since it promotes access to fluorides to all who receive water from water supply systems. The advantages of water fluoridation are that it provides substantial lifelong caries prevention, is cost-effective, and reduces health inequalities: it reaches a substantial number of people worldwide [53].

Thus, it is possible to agree with Buzalaf et al. [18] that reinforce the belief in the importance of the implementation and maintenance of external control of fluoride in water supplies to improve the consistency of water fluoridation. This measure is fundamental to achieve the maximum benefits of water fluoridation, which contributes to improve the oral health condition of people who drink water from those supplies.

This review presented some methodological limitations. Most studies selected used samples collected in different ways (sample size, frequency, storage) and with different criteria, producing high heterogeneity of results. This might have affected the analysis of fluoride concentration in the water. Thus, the high and unexplained heterogeneity observed in the analyzes is an important limitation that must be considered when interpreting our results, which prevents us from being more emphatic in our conclusions. Also, the studies included in this review cover only three of the five Brazilian regions, reflecting the lower coverage of fluoridated water in the north and midwest regions. Therefore, new studies are encouraged to monitor the amount of fluoride in public water supplies in the whole Brazilian territory, so that public policies may be developed and correctly directed to the population. Moreover, standardized studies on fluoride external control are required to produce comparable results in different locations of the country [51]. In this aspect, the implementation of the Vigiflúor system represents a major step towards the surveillance of the public water supply in Brazil [54].

Nevertheless, this study is original and contributed to the development of scientific knowledge from two main points. First, it is the first systematic review with a meta-analysis that assesses the fluoride external control concentration in Brazilian public water supplies. Second, an extensive search strategy was applied without any restriction of language or publication date and including the "grey literature" to avoid selection and publication biases.

## Conclusion

We conclude that the fluoride levels in the public water supply in several Brazilian cities are inadequate to guarantee the anticaries benefits and safety from fluorosis.

The establishment of effective local policies of oral health surveillance is imperative to ensure that the fluoride concentration in the water supply is ideal to guarantee the effectiveness of the fluoride ion and the low risk of fluorosis, thus including the understanding of the epidemiological dynamics of dental caries in the cities.

## Supplementary Information

**Additional file 1. Table S1**. Articles excluded and reasons for exclusion (n = 30).

**Additional file 2. Table S2**. Analysis of publication bias.

**Additional file 3. Figure S1**. Diagonal values of the hat matrix to identify studies that might be outliers or influential for the pooled estimates.

## Data Availability

All data generated or analyzed during this study are included in this published article [and its supplementary information files].

## Abbreviations

PRISMA: Preferred Reporting Items for Systematic Reviews and Meta-Analyses
JBI: The Joanna Briggs Institute
