# Supplementary material for: External control of fluoridation in the public water supplies of Brazilian cities as a strategy against caries: a systematic review and meta-analysis
Source: BMC Oral Health. 2021 Aug 19;21:410. doi: 10.1186/s12903-021-01754-2 (PMC8377988; doi:10.1186/s12903-021-01754-2)
Supplement: Supplementary file 1 — Additional file 1. Table S1. Articles excluded and reasons for exclusion (n = 30). [file 12903_2021_1754_MOESM1_ESM.docx]

**Supplementary Table 1** – Articles excluded and reasons for exclusion (n=30).

| Reference | Author, year | Reason for exclusion | Reference |
| --- | --- | --- | --- |
| 1 | Spadaro et al., 1990 | 2 | Spadaro AC, Polizello AC, Carlomagno DN, Alves LA, Lima SN. Avaliação do teor de fluoreto na água de abastecimento de cidades na região de Ribeirão Preto. Rev Odontol Univ Sao Paulo. 1990;4(3):252–5. Portuguese. |
| 2 | Armonia et al., 1995 | 5 | Armonia PL, Adde CA, Tortamano N, Melo JAJ. Estado atual da fluoração das águas de abastecimento público no município de São Paulo. J. Health Sci. Inst. 1995;13(2):63-6. Portuguese. |
| 3 | Modesto et al., 1999 | 2 | Modesto A, Tanaka FHR, Freitas AD, Cury JA. Avaliaçäo da concentraçäo de fluoreto na água de abastecimenhto público do Município do Rio de Janeiro [Evaluation of the concentration of fluoride in Rio de Janeiro’s public water supply]. Rev Bras Odontol. 1999;56(5):217–21. Portuguese. |
| 4 | D’Aguila et al., 2000 | 3 | d'Aguila PS, Roque OC, Miranda CA, Ferreira AP. Avaliação da qualidade de água para abastecimento público do Município de Nova Iguaçu [Quality assessment of the public water supply in Nova Iguaçu, Rio de Janeiro]. Cad Saude Publica. 2000;16(3):791-798. Portuguese. |
| 5 | Pires et al., 2002 | 5 | Pires LD, Macêdo JABde, Rocha HVA, Lima DC, Vaz UP, Oliveira RFde. Determinação do índice de fluoreto, em águas de abastecimento público na cidade de Juiz de Fora, MG. Hig. Alimente. 2002;16(96):43-50. |
| 6 | Ramires et al., 2004 | 4 | Ramires I, Grec RHC, Cattan L, Moura PG, Lauris JRP, Buzalaf MAR. Avaliação da concentração de flúor e do consumo de água mineral [Evaluation of the fluoride concentration and consumption of mineral water]. Rev. Saúde Pública 2004;38(3):459-465. Portuguese, English. |
| 7 | Vidal et al., 2006 | 2 | Vidal SG, Tovo MF, Kramer PF, Ruschel HC, Ferreira SH. Heterocontrole da fluoretação da água de abastecimento público do município de Torres/RS. Stomatos. 2006;12(22):5–9. Portuguese. |
| 8 | Ramirez et al., 2006a | 2 | Ramires I, Olympio KP, Maria AG, Pessan JP, Cardoso VE, Lodi CS, Buzalaf MA. Fluoridation of the public water supply and prevalence of dental fluorosis in a peripheral district of the municipality of Bauru, SP. J Appl Oral Sci. 2006;14(2):136-41. |
| 9 | Ramirez et al., 2006b | 6 | Ramires I, Maia LP, Rigolizzo DS, Lauris JRP, Buzalaf MAR. Heterocontrole da fluoretação da água de abastecimento público em Bauru, SP, Brasil [External control over the fluoridation of the public water supply in Bauru, SP, Brazil]. Rev Saúde Pública 2006;40(5):883-9. Portuguese, English. |
| 10 | Toassi, 2007 | 2 | Toassi RFC, Kuhnen M, Cislaghi GA, Bernardo JR. Heterocontrole da fluoretação da água de abastecimento público de Lages, Santa Catarina, Brasil [External control of fluoride levels in the public water supply in Lages, Santa Catarina State, Brazil]. Cien Saude Colet. 2007;12(3):727-32. Portuguese. |
| 11 | Catani et al., 2008 | 7 | Catani DB, Amaral RC do, Oliveira C de, Sousa M da LR de, Cury JA. Dez anos de acompanhamento do heterocontrole da fluoretação da água feito por municípios brasileiros, Brasil, 1996-2006. RGO. 2008;56(2):151–5. Portuguese. |
| 12 | Daré et al., 2009 | 6 | Daré F, Sobrinho MD, Libânio M. Avaliação do processo de fluoretação nos sistemas de abastecimento de água da região de Araçatuba, São Paulo [Evaluation of the fluoridation in the water supply systems of Araçatuba, São Paulo, Brazil]. Eng Sanit Ambient 2009;14(2):173-182. Portuguese. |
| 13 | Silva et al., 2011 | 3 | Silva RA, Petrarca MH, Santos RC, Yamamoto IT, Marques LRM. Monitoramento da concentração de íons fluoreto na água destinada ao consumo humano proveniente de um município com diferentes sistemas de abastecimento público [Monitoring the fluoride ions concentration in water for human consumption from a municipality with different public water supply systems]. Rev Inst Adolfo Lutz. 2011;70(2):220-4. Portuguese. |
| 14 | Frazão et al., 2011 | 1 | Frazão P, Peres MA, Cury JA. Drinking water quality and fluoride concentration. Rev Saude Publica. 2011;45(5):964-73. |
| 15 | Olivati et al., 2011 | 6 | Olivati FN, Souza MLR, Tenuta LMA, Cury JA. Quality of drinking water fluoridation of Capão Bonito, SP, Brazil, evaluated by operational and external controls. Rev Odonto Cienc 2011;26(4):285-290. |
| 16 | Motter et al., 2011 | 7 | Motter J, Moyses ST, França BHS, Carvalho ML de, Moysés SJ. Análise da concentração de flúor na água em Curitiba, Brasil: comparação entre técnicas [Analysis of water fluoride concentration in Curitiba, Brazil: comparison of techniques]. Rev Panam Salud Publica. 2011;29(2):120–5. Portuguese. |
| 17 | Scorsafava et al., 2011 | 3 | Scorsfava MA, Souza Ade, Sakuma H, Stofer M, Nunes CA, Milanez TV. Avaliação da qualidade da água de abastecimento no período 2007- 2009. Rev. Inst. Adolfo Lutz. 2011;70(3):395-403. Portuguese. |
| 18 | Moimaz et al., 2012 | 6 | Moimaz SAS, Saliba O, Chiba FY, Sumida DH, Garbin SAS, Saliba NA. Fluoride Concentration in Public Water Supply: 72 Months of Analysis. Braz Dent J 2012;23(4): 451-456. |
| 19 | Paredes et al., 2012 | 2 | Paredes SO, Sampaio FC, Forte FDS. Levels of natural fluoride in the public water supply of São Luís, Maranhão, Brazil. Rev. Odont. Cienc. 2012;27(4):304-8. |
| 20 | Soares et al., 2013 | 7 | Soares Carlos Cesar da Silva. Vigilância da fluoretação da água de abastecimento público no Município de São Paulo, no período 1990-2011 [Dissertação on the Internet]. São Paulo: Faculdade de Saúde Pública, Universidade de São Paulo; 2013 [cited 2020 Aug 10]. 163 p. DOI 10.11606/D.6.2013.tde-16122013-135053. Available from: <https://www.teses.usp.br/teses/disponiveis/6/6135/tde-16122013-135053/publico/CarlosCesar.pdf>. Portuguese. |
| 21 | Buzalaf et al., 2013 | 6 | Buzalaf MA, Moraes CM, Olympio KP, Pessan JP, Grizzo LT et al. Seven years of external control of fluoride levels in the public water supply in Bauru, São Paulo, Brazil. J Appl Oral Sci. 2013;21(1):92. |
| 22 | Silva et al., 2013 | 5 | Silva MAM, Lima FRGS, Queiroz JPL, Santos G, Cardoso CE. O teor de fluoretos na água de consumo no ambiente escola e a perspectiva de controle da cárie dentária [The fluoride content of drinking water in the school environment and the prospect of controlling dental caries]. Rev. APS. 2013;16(4):429-36. Portuguese. |
| 23 | Paredes et al., 2014 | 6 | Paredes SO, Sampaio FC, Forte FDS. External Control over Fluoridation of the Public Water Supply in São Luís, MA, Brazil. Brazilian Research in Pediatric Dentistry and Integrated Clinic 2014;14(2):129-140. |
| 24 | Dovidauskas et al., 2017 | 5 | Dovidauskas S, Okada IA, Iha MH, Cavallini AG, Okada MM, Briganti RC. Quality Assessment of fluoridation of public water supply in 88 municipalities in the Northeast region of the state of São Paulo (Brazil). Vigil. Sanit. Debate. 2017;5(3):1-10. |
| 25 | Moimaz et al., 2017 | 2 | Moimaz SAS, Santos LFP, Saliba NA, Saliba O. Eleven years of monitoring fluoride content in public water supplies: methods, products, and importance to public health. Biosci. j. 2017;33(3):799-808. |
| 26 | Belotti et al., 2018 | 3 | Belotti L, Frazao P, Esposti CDD, Cury JA, Santos Neto ET, Pacheco KTS. Quality of the water fluoridation and municipal-level indicators in a Brazilian metropolitan region. Rev. Ambient. Água. 2018;13(6):e2270. |
| 27 | Romani et al., 2018 | 7 | Romani CD, Stancari SCA, Nascentes GAN, Anversa L. Public water supply fluoridation: 10 years of monitoring in 38 municipalities of Centro-Oeste Paulista, São Paulo, Brazil. Vigil. sanit. debate 2018;6(4):47-55 |
| 28 | Scalize et al., 2018 | 2 | Scalize PS, Pinheiro RVN, Ruggeri Junior HC, Albuquerque A, Lobón GS, Arruda PN. Heterocontrole da fluoretação da água de abastecimento público em cidades do estado de Goiás, Brasil [External control of fluoridation in public water supply systems in the state of Goiás, Brazil]. Cien Saude Colet. 2018;23(11):3849-3860. Portuguese, English. |
| 29 | Meschede et al., 2018 | 2 | Meschede MSC, Figueiredo BR, Alves RIS, Segura-Muñoz SI. Drinking water quality in schools of the Santarém region, Amazon, Brazil, and health implications for school children. Rev. Ambient. Água. 2018;13(6):e2218. |
| 30 | Moimaz et al., 2020 | 6 | Moimaz SAS, Santos LFPD, Saliba TA, Saliba NA, Saliba O. Vigilância em saúde: fluoretação das águas de abastecimento público em 40 municípios do estado de São Paulo, Brasil [Health surveillance: public water supply fluoridation in 40 municipalities of São Paulo, Brazil]. Cien Saude Colet. 2020 Jul 8;25(7):2653-2662. Portuguese, English. |

**Legend**: 1) Review article; 2) Did not perform external control for the minimum time (12 months); 3) Out of scope; 4) Did not use public supply water; 5) Did not use the electrometric method; 6) articles that used artificially and naturally fluoridated water; 7) Used secondary data.
