# Supplementary material for: External control of fluoridation in the public water supplies of Brazilian cities as a strategy against caries: a systematic review and meta-analysis
Source: BMC Oral Health. 2021 Aug 19;21:410. doi: 10.1186/s12903-021-01754-2 (PMC8377988; doi:10.1186/s12903-021-01754-2)
Supplement: Supplementary file 2 — Additional file 2. Table S2. Analysis of publication bias. [file 12903_2021_1754_MOESM2_ESM.docx]

**Supplementary Table 2** – Analysis of publication bias.

|  | **Number of studies** | **Bias** | **Standard error** | **P value** |
| --- | --- | --- | --- | --- |
| **Samples outside the acceptable range** | | |  |  |
| **Overall effect** | 20 | 3.13 | 2.34 | 0.198 |
| **Region** |  |  |  |  |
| Northeast | 5 | -6.76 | 0.66 | 0.002 |
| Southeast | 6 | -1.29 | 4.07 | 0.768 |
| South | 9 | -1.89 | 2.96 | 0.543 |
| **Samples below the acceptable range** | | |  |  |
| **Overall effect** | 15 | 5.67 | 3.66 | 0.145 |
| **Region** |  |  |  |  |
| Northeast | 5 | -7.31 | 0.33 | < 0.001 |
| Southeast | 6 | 0.67 | 5.04 | 0.901 |
| South | 4 | 11.90 | 1.37 | 0.013 |
| **Samples above the acceptable range** | | |  |  |
| **Overall effect** | 15 | -1.80 | 1.54 | 0.262 |
| **Region** |  |  |  |  |
| Northeast | 5 | 0.55 | 0.33 | 0.195 |
| Southeast | 6 | -1.96 | 2.43 | 0.467 |
| South | 4 | -7.39 | 1.77 | 0.053 |
